# Hyaluronic Acid‐Based Bleaching Gels With NF_TiO_2_ and Violet LED: Efficacy and Cytotoxicity of Low‐Concentration H_2_O_2_ for In‐Office Bleaching

**DOI:** 10.1111/jerd.13484

**Published:** 2025-05-07

**Authors:** Marcos Roberto de Lima de Benati, Priscila Borges Gobbo de Melo, Rafael Antonio de Oliveira Ribeiro, Fernando Luis Esteban Florez, Carlos Alberto de Souza Costa, Vanessa Cavalli

**Affiliations:** ^1^ Department of Restorative Dentistry University of Campinas—Piracicaba Dental School (FOP—UNICAMP) Piracicaba SP Brazil; ^2^ Department of Physiology and Pathology Araraquara School of Dentistry (FOAr/UNESP) Araraquara São Paulo Brazil; ^3^ Department of Restorative Sciences University of Oklahoma—Health Sciences Center Oklahoma City Oklahoma USA

**Keywords:** dental bleaching, hyaluronic acid, hydrogen peroxide, nanotechnology, titanium

## Abstract

**Objective:**

To evaluate the efficacy and cytotoxicity of in‐office bleaching gels with hyaluronic acid (HA) or carbomer 940 (CAR), titanium dioxide nanoparticles co‐doped with nitrogen and fluorine (NF_TiO_2_), and hydrogen peroxide (HP; H_2_O_2_) at 1.5% and 6% with violet LED irradiation.

**Materials and Methods:**

48 bovine enamel/dentin discs (5 × 3 mm) stained with black tea for 24 h were assigned to six groups (*n* = 8): HA‐1.5%HP + LED, HA‐6%HP + LED, CAR‐1.5%HP + LED, CAR‐6%HP + LED, 35%HP‐commercial (control), and a negative control (no treatment). The discs were placed in artificial pulp chambers (APCs) and underwent three 30‐min bleaching sessions with 20 violet LED cycles (1‐min activation, 30‐s pause) at 7‐day intervals. Extracts were applied to MDPC‐23 cells, assessing color change (ΔE_00_), whiteness index (ΔWI_D_), H_2_O_2_ diffusion, cell viability (CV), oxidative stress (OxS), and cell morphology (SEM). Data were analyzed by one‐way ANOVA and Tukey post hoc test (*α* = 0.05).

**Results:**

Gels with HA showed no statistical difference in ΔE_00_ and ΔWI_D_ compared with 35%HP‐commercial (*p* > 0.05). H_2_O_2_ diffusion and oxidative stress were lower in 1.5% and 6% HP groups. Cell viability was higher in 1.5% HP groups (*p* < 0.05). There were no changes in cell morphology.

**Conclusion:**

Bleaching gels with HA, NF_TiO_2_ NPs, low H_2_O_2_ concentrations, and violet LED irradiation reduced cytotoxicity without compromising efficacy.

**Clinical Relevance:**

Experimental bleaching gels with hyaluronic acid, NF_TiO_2_ nanoparticles, low H_2_O_2_ concentrations, and combined with violet LED irradiation achieve similar efficacy to high‐H_2_O_2_ gels (35%). This approach also promises to reduce cytotoxic damage, providing a safer in‐office bleaching option.

## 1 Introduction

Tooth bleaching is a common procedure in dental practice due to its ability to produce a rapid and effective change in tooth color [1, 2, 3]. High concentrations of hydrogen peroxide (HP; H_2_O_2_) used during in‐office dental bleaching procedures, typically ranging from 25% to 40%, are the primary cause of side effects on dental enamel associated with bleaching treatments [4, 5]. Commonly reported adverse effects include increased surface roughness [6], alterations in mineral composition (Ca/P ratio) and substrate morphology [3, 7], as well as reduction in enamel microhardness [7, 8]. Moreover, the dissociation of H_2_O_2_ generates reactive oxygen species (ROS), which diffuse through dentinal tubules to break down pigment molecules [9]. Higher concentrations of the bleaching agent facilitate the penetration of ROS into dentin, reaching the pulp tissue and inducing cytotoxic effects on pulp cells [10, 11, 12, 13]. Cytotoxicity, an adverse biological response, clinically manifests as tooth sensitivity, a frequently reported symptom during and/or after the bleaching procedure [14].

According to the literature, the prevalence of tooth sensitivity ranges from 50% to 93% among patients undergoing in‐office bleaching treatments, with reported pain intensity averaging 2.8 on a scale of 0 to 4 [15, 16]. Studies have demonstrated that higher concentrations of H_2_O_2_ are associated with increased oxidative stress in pulp cells, initiating an inflammatory response that can ultimately lead to cell death [17, 18, 19]. Consequently, recent advancements in dental bleaching have focused on optimizing treatment protocols. Proposed modifications include incorporating catalysts to enhance H_2_O_2_ efficacy [20, 21], irradiating bleaching gels with light [22, 23], and modifying the composition of traditional thickening agents [24, 25, 26]. These strategies aim to reduce H_2_O_2_ concentrations, thereby minimizing its harmful effects on enamel properties and cytotoxicity while maintaining the overall bleaching efficacy of the treatment.

Titanium dioxide (TiO_2_) is utilized in the biomedical field due to its biocompatible and antimicrobial properties [27, 28, 29, 30]. In dental bleaching, it enhances stain removal when activated by light [20]. Previous studies have demonstrated that gels containing nitrogen‐doped TiO_2_ nanoparticles (N_TiO_2_) [31] or co‐doped with nitrogen and fluorine (NF_TiO_2_) [32] can significantly enhance the color change achieved by bleaching agents. Co‐doping with nitrogen and fluorine modifies the electronic structure of TiO_2_, enabling the nanoparticles to absorb light within the visible spectrum [33]. When exposed to violet LED (405–410 nm) and combined with lower concentrations of H_2_O_2_, these nanoparticles function as semiconductors, potentially increasing the production of ROS, particularly hydroxyl radicals (OH‐), which have longer half‐lives and effectively break down organic chromophore compounds in the enamel [32, 34, 35].

The specific emission wavelength of violet light corresponds to the absorption spectrum of chromophore molecules in dental enamel, rendering these molecules particularly susceptible to photosensitization at this wavelength [36]. Although violet light induces clinically perceptible color changes, removing pigments occurs primarily on an extrinsic level, affecting only the chromophores within the enamel [37]. Evidence from in vitro studies [38, 39] and clinical trials [40, 41] demonstrates that violet light enhances the bleaching efficacy of low‐concentration peroxides. Furthermore, the incorporation of NF_TiO_2_ nanoparticles into low‐concentration H_2_O_2_ formulations amplifies this effect, providing a safer alternative to conventional bleaching protocols that rely on 35% hydrogen peroxide [20, 23].

In addition to the active component, bleaching gels typically contain carbomer 940 (CAR), a synthetic water‐soluble polyacrylic acid polymer that acts as a vehicle for the release of ROS on the tooth surface. However, this polymer exhibits pH variations, leading to alterations in the morphology, composition, and structure of dental enamel [32, 42]. Hyaluronic acid (HA) is a naturally occurring polysaccharide found in the extracellular matrix of connective tissues in the human body [43]. It is a widely recognized biopolymer in healthcare, valued for its viscoelastic properties, physiological activity, biocompatibility, biodegradability, and bioactivity [44, 45]. Due to these characteristics, HA hydrogel could contribute to the chemical stability of H_2_O_2_, influencing its ability to modulate the release of hydrogen peroxide. This controlled diffusion of the bleaching agent would reduce adverse effects, such as cellular cytotoxicity and consequent postoperative sensitivity. In this context, the development of biopolymer‐based hydrogels with optimized rheological properties that create a favorable molecular environment could improve ROS movement and enhance the bleaching effect.

Thus, this study aimed to evaluate the bleaching efficacy and cytotoxicity of experimental CAR or HA‐based bleaching gels containing 5 wt% NF_TiO_2_ nanoparticles combined with low concentrations of H_2_O_2_ (1.5% and 6%) and irradiated with violet LED. The research hypotheses tested were that the experimental gels: (1) would not achieve the same bleaching efficacy as commercial gels containing 35% hydrogen peroxide, and (2) would not be cytotoxic to MDPC‐23 pulp cells.

## 2 Materials and Methods

The experimental gels were formulated with HA (Sigma Aldrich Chemical, St. Louis, MO, USA) or CAR (Sigma Aldrich Chemical, St. Louis, MO, USA) and incorporated with 5 wt% NF_TiO_2_ nanoparticles. Bovine enamel‐dentin blocks were sectioned and stained with black tea (Dr. Oetker, Sao Paulo—SP, Brazil). The samples were selected based on their initial color analysis (T_0_) (*n* = 8), randomized into experimental groups, and subjected to bleaching with the experimental gels associated with violet LED light. The response variables studied were bleaching efficacy (ΔE_00_ and ΔWI_D_) and cytotoxicity, including cell viability analysis (CV), H_2_O_2_ diffusion, oxidative stress (OxS), and cell morphology (SEM). The groups were divided according to the thickening agent and the hydrogen peroxide concentration, as represented in Table 1.

**TABLE 1:** Experimental Design.

Experimental units	Bovine enamel/MDPC‐23 odontoblastic cells	
Parameters under study	1. Thickeners	Hyaluronic acid (HA); Carbomer 940 (CAR).
	2. Hydrogen peroxide concentration	HP 1.5%; HP 6%.

Table 2 presents the composition and specification of each material used in the formulation of the experimental bleaching gels. It describes the components employed, their respective concentrations, and specific characteristics, providing a detailed understanding of the formulations tested in the study.

**TABLE 2:** Composition of the experimental gels used in the study.

Experimental gels	Experimental materials	Specification/Composition
HA‐gels	Hyaluronic acid‐based hydrogel used as a thickening agent	Hyaluronic acid (Sigma‐Aldrich, St. Louis, MI, USA) 2%, ultrapure water and potassium hydroxide
	Co‐doped titanium dioxide nanoparticles (NF_TiO_2_) (5%)	Ti (OBu)_4_ (Aldrich, 97%), C_2_H_5_OH (200‐proof Decon Labs, King of Prussia, PA, EUA), C_18_H_35_NH_2_ (Aldrich, 70%), C_18_H_34_O_2_ (Aldrich, 90%), NH_4_F (based on Ti content; crystalline, ACS, Alfa Aesar), and ethanol‐water solution.
	Hydrogen peroxide solution	1.5 or 6% hydrogen peroxide solution (Sigma‐Aldrich, St. Louis, MI, USA)
CAR‐gels	Carbomer 940‐based hydrogel used as a thickening agent	Carbomer 940 (Sigma‐Aldrich, St. Louis, MI, USA) 2%, ultrapure water and potassium hydroxide
	Co‐doped titanium dioxide nanoparticles (NF_TiO_2_) (5%)	Ti (OBu)_4_ (Aldrich, 97%), C_2_H_5_OH (200‐proof Decon Labs, King of Prussia, PA, EUA), C_18_H_35_NH_2_ (Aldrich, 70%), C_18_H_34_O_2_ (Aldrich, 90%), NH_4_F (based on Ti content; crystalline, ACS, Alfa Aesar), and ethanol‐water solution
	Hydrogen peroxide solution	1.5 or 6% hydrogen peroxide solution (Sigma‐Aldrich, St. Louis, MI, USA)

The control group (35%HP‐commercial) was treated with a commercial bleaching gel (Whiteness HP, FGM, Joinville, SC, Brazil) and was not irradiated, following the manufacturer's recommendations (Composition: 35% hydrogen peroxide, glycerol, inert filler and deionized water; pH reported by the manufacturer: 7.0). The negative control group (no treatment) was used only in cell viability and oxidative stress analyses to represent baseline (100%) values.

Based on the initial color analysis before bleaching (T_0_), the samples were randomized into six groups (*n* = 8) using the block randomization method based on the L* values [20].

- HA‐1.5% HP + LED
- HA‐6% HP + LED
- CAR‐1.5% HP + LED
- CAR‐6% HP + LED
- 35% HP‐commercial
- Negative control (no treatment)

### 2.1 Synthesis of NF_TiO_2_ Nanoparticles

NF_TiO_2_ nanoparticles were synthesized using solvothermal processes according to the protocol previously described by Esteban Florez et al. [33]. A solution composed of 1.7 g of TiO (IV‐butoxide, Sigma‐Aldrich, 97%), 4.6 g of ethanol (Decon Labs), 6.8 g of oleylamine (Sigma‐Aldrich, 70%), and 7.1 g of oleic acid (Sigma‐Aldrich, 90%) was prepared and mixed with 20 mL of 4% hydrated alcohol (18‐MΩ Milli‐Q, Decon Labs). The solution was placed in a high‐pressure reaction vessel (Paar Series 5000 Multiple Reactor System, Moline, Illinois) at 180°C for 24 h. The vessels were stirred in an external magnetic field using stir bars. After cooling, the solutions were decanted and rinsed three times with anhydrous ethanol to remove surfactants. The pure TiO_2_ NPs were immediately stored in 20 mL of ethanol and centrifuged for 15 min at 8.000 rpm.

### 2.2 Preparation of Experimental Gels

HA (Sigma Aldrich Chemical, St. Louis, MO, USA) and CAR (Sigma Aldrich Chemical, St. Louis, MO, USA), the polymers used as a matrix for the bleaching gels, were diluted in distilled water and homogenized in a mixer homogenizer (Speed Mixer, Landrum, SC, USA) until the viscosity that ensured stable permanence in the tooth structure without slipping was achieved. The final concentrations of HA and CAR used in the gels were both 2%.

The NF_TiO_2_ nanoparticles in alcohol were mixed for 30 s, and aliquots corresponding to the intended concentrations (5%) were centrifuged. The ethanol was almost completely removed, and the nanoparticles were added to the hydrogel and homogenized. Subsequently, a KOH solution was added to the hydrogel to neutralize the mixture using a pH meter coupled to a microelectrode (DG‐101SC, Mettler Toledo, Brazil), and the pH of all gels was standardized at 6.0, while the Whiteness HP (FGM, Dental Industry and Commerce, Joinville, PR, Brazil) exhibited a mean pH of 7.1. Hydrogen peroxide (35% Merck) was diluted to concentrations of 1.5% and 6% and then added to the HA or CAR hydrogel in a 2:1 ratio.

### 2.3 Preparation and Selection of Samples

Bovine teeth with intact enamel surfaces, free from fractures or cracks, were selected, cleaned, and disinfected in a 0.5% thymol solution (Labsynth, Diadema, SP, Brazil). Discs of enamel/dentin with a diameter of 5 mm and a thickness of 3 mm were obtained using a bench drill (FSB 16, Pratika, Schulz) and crown drill bits. The dentin surface of the samples was polished (AROTEC, São Paulo, Brazil) and leveled with #600 grit sandpaper to ensure parallelism and achieve a standardized total thickness of 2.3 mm^11^. The enamel surfaces were not polished.

The staining protocol was based on the study by Matos et al. [23]. The samples were isolated with a nail polish base, leaving only the vestibular enamel exposed, and were immersed in a black tea solution with neutralized pH (7.0) (2 g of black tea in 100 mL of boiled distilled water for 4 h). After staining, the samples were cleaned with pumice stone (SS White, São Paulo, Brazil) and a Robson brush to remove non‐adhered particles and were stored in artificial saliva (1.5 mM Ca; 0.9 mM PO4 and 150 mM KCl in a 20 mM tris buffer solution, pH 7.0) in an incubator at 37°C for 7 days to stabilize the color before the treatments, with the artificial saliva being replaced every 2 days.

The enamel/dentin discs were adapted into artificial pulp chambers (APCs) using two silicone rings, which were sealed with utility wax to prevent any leakage of the bleaching gel into the pulp space. The disc and APC assemblies were positioned in 24‐well plates (KASVI Imp.), ensuring that only the dentin was in contact with the culture medium, while the enamel surface remained exposed for the bleaching protocol. The device + disc assembly was sterilized using ethylene oxide (ACECIL—Centro de Esterilização Com. Ind. Ltda, Campinas, São Paulo, Brazil). Immediately after the completion of the bleaching procedures, following the 30‐min protocol, the culture medium in contact with the dentin was collected, homogenized, and distributed into 24 or 96‐well plates (KASVI Imp.), respectively, where pulp cells had been previously cultured. The extracts were incubated for 1 h in contact with the cells, which were then subjected to the analyses described below.

### 2.4 Bleaching Protocol

In the groups treated with violet LED light, the bleaching gels were applied to the buccal surface of the enamel for 30 min, with a single application per session. Irradiation with the violet LED was initiated immediately after the gel application. The LED Bright Max Whitening device—BMW (MMOptics, São Carlos, SP, Brazil) was used, with a wavelength of 401.82 nm (violet) and a power of 1.2 W for all groups. The procedure consisted of 20 cycles of 1‐min irradiation, with 30‐s intervals between cycles. After irradiation, the gel was removed using a high‐power suction device, and the surface was thoroughly rinsed with distilled water. This protocol was repeated over 3 sessions, with 7‐day intervals between each session.

In the groups that did not receive violet LED light irradiation, the bleaching gels were similarly applied to the buccal surface of the enamel for 30 min, with a single application per session. Afterward, the gel was removed using a high‐power suction device and rinsed thoroughly with distilled water. The protocol consisted of 3 applications, with 7‐day intervals between sessions [32].

### 2.5 Color Evaluation (ΔE_00_ e ΔWI_D_ )

A digital spectrophotometer (EasyShade, Vita Zahnfabrik, Bad Säckingen, Germany) determined the color parameters L* (black–white axis), a* (red‐green axis), and b* (yellow‐blue axis). Two color measurements were taken: before bleaching (T_0_) and 14 days after the last bleaching session (T_1_). Three readings were performed on each sample in different positions, rotating the specimen between each reading, and the average was calculated. Color change was evaluated using the CIEDE2000 formula [ΔE_00_ = (ΔL0/KLSL)^2^ + (ΔC0/KCSC)^2^ + (ΔH0/KHSH)^2^ + RT*(ΔC0/KCSC) (ΔH0/KHSH)^1/2^]. The whiteness index was calculated using the following formula: WI_D_ = (0.511 × L)— (2.324 × a*)—(1.100 × b*). These equations were calculated in T_0_ and T_1_. ΔE_00_ and ΔWI_D_ refer to the final color obtained by the value of T_1_−T_0_ [1, 2].

### 2.6 Culture of Odontoblastic MDPC‐23 Cells

MDPC‐23 immortalized odontoblastic cell line cells, stored in liquid nitrogen at the Laboratory of Experimental Pathology and Biomaterials at the Araraquara School of Dentistry—UNESP, were thawed and cultured in 75 cm^2^ flasks (KASVI Imp., São José dos Pinhais, PR, Brazil). Cell passages were performed every 3 days until enough cells were obtained. The pulp cells were then cultivated in Dulbecco's Modified Eagle's Medium (DMEM; GIBCO, Grand Island, NY, USA) supplemented with 10% fetal bovine serum (FBS; GIBCO), along with 100 IU/mL of penicillin and 100 μg/mL of streptomycin, and 2 mmol/L of glutamine (GIBCO).

The plates containing the cells were maintained in a humidified atmosphere at 37°C, with 5% CO_2_ and 95% air. The cells were plated and counted using an inverted light microscope and will be replicated until an adequate number of cells is obtained [5].

### 2.7 H_2_O_2_ Diffusion

Aliquots of 100 μL from each group's extracts (*n* = 8) were transferred into tubes containing 900 μL of acetate buffer solution (2 mol/L, pH 4.5), which stabilizes the pH. Subsequently, 500 μL of this solution was transferred into tubes containing water and violet leucocrystal dye (0.5 mg/mL; Sigma). After thorough mixing, 50 μL of horseradish peroxidase enzyme solution (1 mg/mL, Sigma) was added to these tubes. The absorbance of the solutions was measured using a spectrophotometer at a wavelength of 596 nm. A standard curve generated from known concentrations of H_2_O_2_ was employed to convert the optical density values obtained from the samples into micrograms of H_2_O_2_ per mL of extract [19].

### 2.8 Cell Viability (CV)

For this test, 10% Alamar Blue (Life Technologies; Grand Island, NY, USA) solution was prepared in DMEM without FBS. Then, 500 μL was distributed to each well containing the extracts, and the plates were incubated with 5% CO_2_ at 37°C for 4 h. The oxidized form of Alamar Blue presents a blue color, which is converted into its reduced form (a pink color) due to mitochondrial activity. Two hundred μL of each sample (extract) was transferred to a 96‐well plate in order to determine the mitochondrial activity by measuring the fluorescence of the reduced salt at a spectrophotometer (excitation at 530–560 nm; emission at 590 nm, Synergy H1) [20]. These fluorescence values were converted into percentages by normalization using the mean of the negative control group.

### 2.9 Oxidative Stress (OxS)

Cellular oxidative stress was assessed by estimating the production of ROS by the cells immediately after the bleaching procedure. For this purpose, MDPC‐23 cells previously cultured in sterilized 96‐well plates were exposed to a carboxy‐H_2_DCFDA probe (Invitrogen, San Francisco, CA, USA) at a concentration of 10 μg/mL for 30 min. This probe is cell‐permeable and emits fluorescence upon interaction with ROS. Subsequently, aliquots of 100 μL from the extracts of each group were applied to the cells for 1 h, and the fluorescence intensity was measured at 592 nm excitation and 517 nm emission (Synergy H1) after the exposure period. The data were normalized to the negative control group, as previously described [17].

### 2.10 Cell Morphology (SEM)

For this analysis, the cells were seeded onto glass slides positioned at the bottom of 24‐well plates (*n* = 4), and the same bleaching procedures described earlier were performed. After the incubation period of the cells in contact with the extracts, the media were aspirated, and the MDPC‐23 cells that remained adhered to the glass slides were fixed for 1 h in 1 mL of 2.5% glutaraldehyde (VETEC Química Fina LTDA, Duque de Caxias, RJ, Brazil). Following the initial fixation, the glass slides containing the cells were washed three times with 1 mL of PBS (5 min per wash) and post‐fixed with 200 μL of 1% osmium tetroxide for 1 h. This was followed by two additional washes with 1 mL of PBS for 5 min each. Subsequently, the cells were washed three times with 200 μL of HMDS (1,1,1,3,3,3‐Hexamethyldisilazane; Sigma‐Aldrich Corp., St. Louis, MO, USA) for 20 min each. Finally, the slides were placed onto metal stubs (devices for fixation and analysis in SEM) and kept in a desiccator (Laborquimi, Poá, SP, Brazil) for 72 h, followed by gold coating for analysis via SEM (FEI Inspect S50, Thermo Fisher. Scientific, Hillsboro, Oregon, USA). The samples were evaluated qualitatively, observing their morphology (cytoplasmic contour) and cellular integrity (membrane rupture) at magnifications of 1000× and 4000× [19].

### 2.11 Statistical Analysis

The collected data underwent the Shapiro–Wilk test to assess normality and the Levene test to analyze homoscedasticity, followed by the one‐way analysis of variance (ANOVA) complemented by Tukey, for the quantitative data, and a descriptive analysis for qualitative data. All the statistical analyses performed were based on a level of significance of 5%. The statistical power of the analysis was established by means of SPSS Statistics 26 software (version 26.0, IBM, Chicago, IL, USA).

## 3 Results

### 3.1 Color Evaluation (ΔE_00_ e ΔWI_D_ )

**FIGURE 1:**
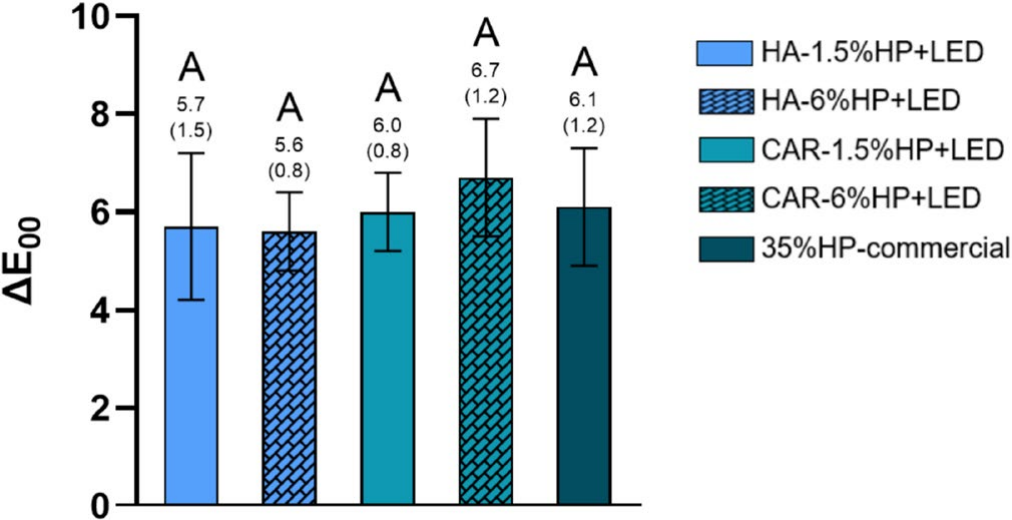
Analysis of ΔE_00_ 14 days after the final bleaching session. Different letters indicate statistically significant differences between groups (One‐way ANOVA; Tukey's test, *α* = 0.05/*n* = 8).

Figure 1 illustrates the color change (ΔE_00_) in the groups treated with experimental gels 14 days after the last bleaching session. All experimental groups exhibited ΔE_00_ values comparable to the control group (35% HP‐commercial), with no statistically significant differences regardless of the thickener type or hydrogen peroxide concentration (*p* > 0.05).

### 3.2 Whiteness Index (ΔWI_D_ )

**FIGURE 2:**
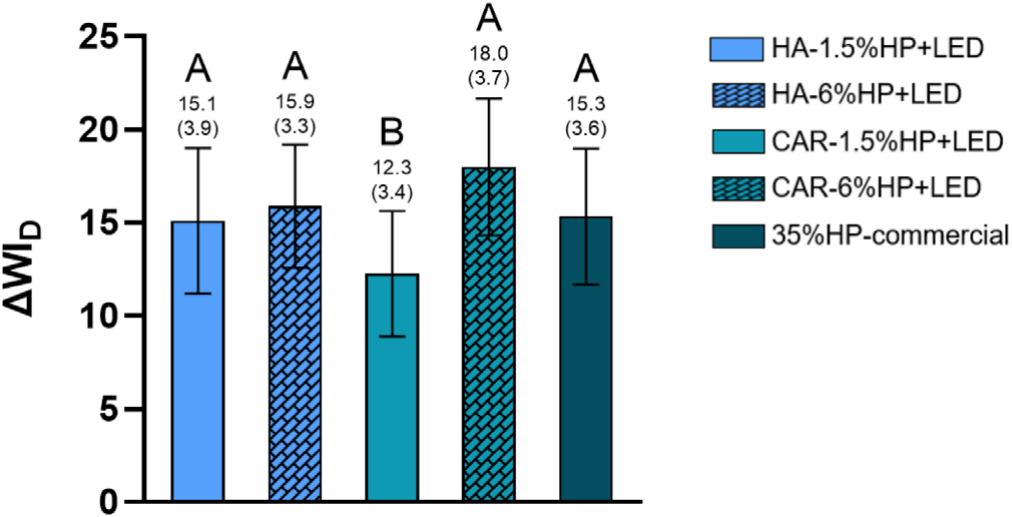
ΔWI_D_ analysis of samples 14 days after the final bleaching session. Distinct letters indicate statistically significant differences between groups (One‐way ANOVA; Tukey's test, *α* = 0.05/*n* = 8).

Figure 2 illustrates the whiteness index (ΔWI_D_) 14 days after the final bleaching session. There was no statistically significant difference between the HA groups and the control group (35%HP‐commercial), regardless of hydrogen peroxide concentration (*p* > 0.05). For the CAR groups, only the CAR‐6%HP + LED group showed no significant difference from the control group (*p* > 0.05).

### 3.3 H_2_O_2_ diffusion

**FIGURE 3:**
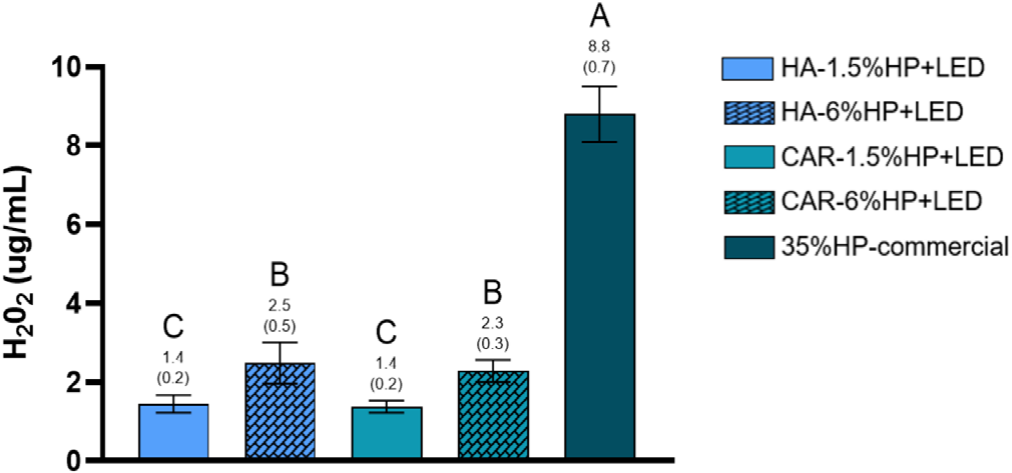
Analysis of H_2_O_2_ diffusion. Bar chart showing the mean values and standard deviations of H_2_O_2_ concentration in the extracts. Different letters indicate statistically significant differences between groups (One‐way ANOVA; Tukey's test, *α* = 0.05/*n* = 8).

Figure 3 shows the concentration of H_2_O_2_ in the extracts after a single bleaching session. The HA‐1.5% HP + LED and CAR‐1.5% HP + LED groups exhibited the lowest concentrations of H_2_O_2_, which were statistically different from the HA‐6% HP + LED and CAR‐6% HP + LED groups (*p* < 0.05), which showed intermediate results. Furthermore, the 35% HP‐commercial control group presented statistically higher values than all experimental groups (*p* < 0.05).

### 3.4 Cell Viability (CV)

**FIGURE 4:**
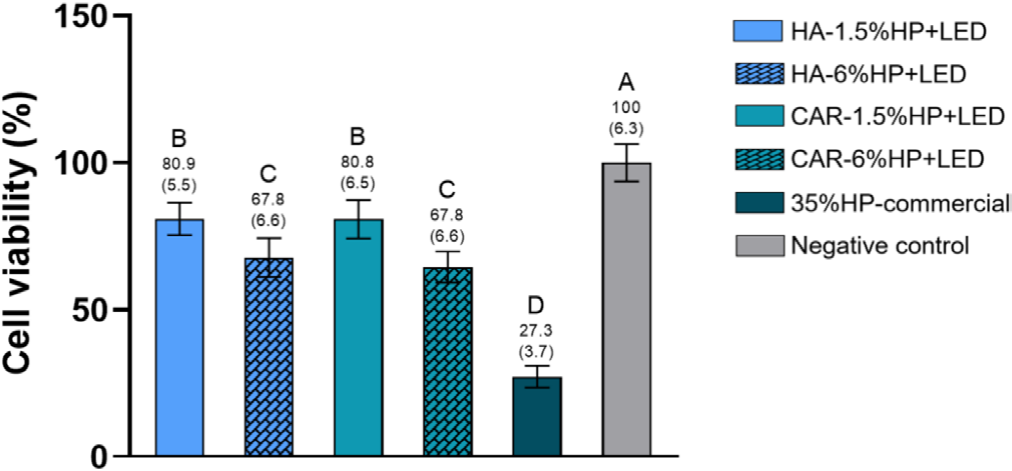
Analysis of cell viability. Figure showing the mean values and standard deviations of cell viability. Different letters indicate statistically significant differences between groups (One‐way ANOVA; Tukey's test, *α* = 0.05/*n* = 8).

Figure 4 presents the CV after a single bleaching session. The negative control group was considered to have 100% cell viability. A decrease in cell viability was observed in all bleached groups. The groups containing 1.5% H_2_O_2_ (HA‐1.5% HP + LED and CAR‐1.5% HP + LED) showed higher CV and were statistically different from the groups containing 6% H_2_O_2_ (*p* < 0.05). The 35%HP‐commercial group showed the lowest CV, statistically different from all other experimental groups (*p* < 0.05).

### 3.5 Oxidative Stress (OxS)

**FIGURE 5:**
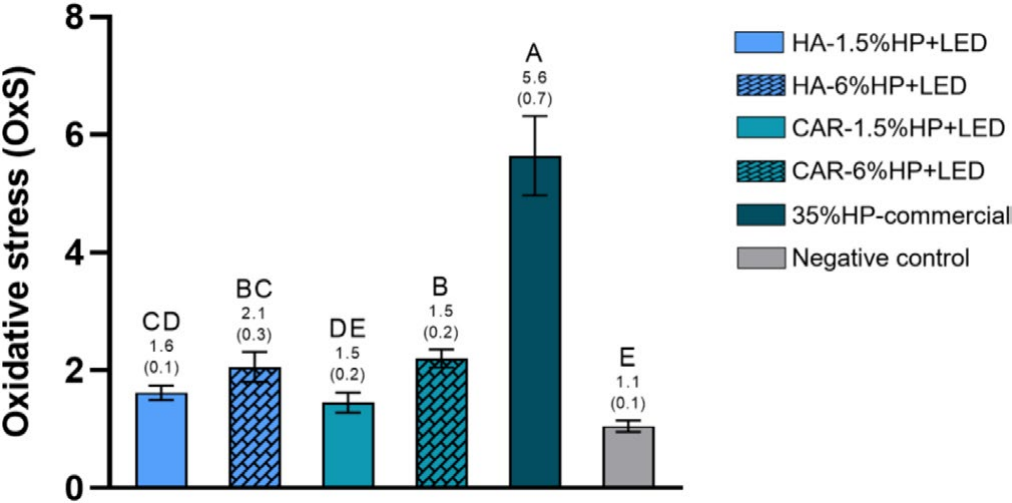
OxS analysis. Figure displaying the mean and standard deviation values of OxS. Different letters indicate statistically significant differences between groups (One‐way ANOVA; Tukey's Test, *α* = 0.05/*n* = 8).

Figure 5 presents the OxS values after a single bleaching session. The OxS of MDPC‐23 cells was lower when the bleaching gels HA‐1.5% HP + LED and CAR‐1.5% HP + LED were used. Despite their low values, they differed statistically from the CAR‐6% HP + LED group (*p* < 0.05). The negative control group exhibited the lowest OxS, with no statistically significant difference from the CAR‐6% HP + LED group, while the highest value was observed in the 35% HP‐commercial group, which differed significantly from all other study groups (*p* < 0.05).

### 3.6 Cell Morphology (SEM)

**FIGURE 6:**
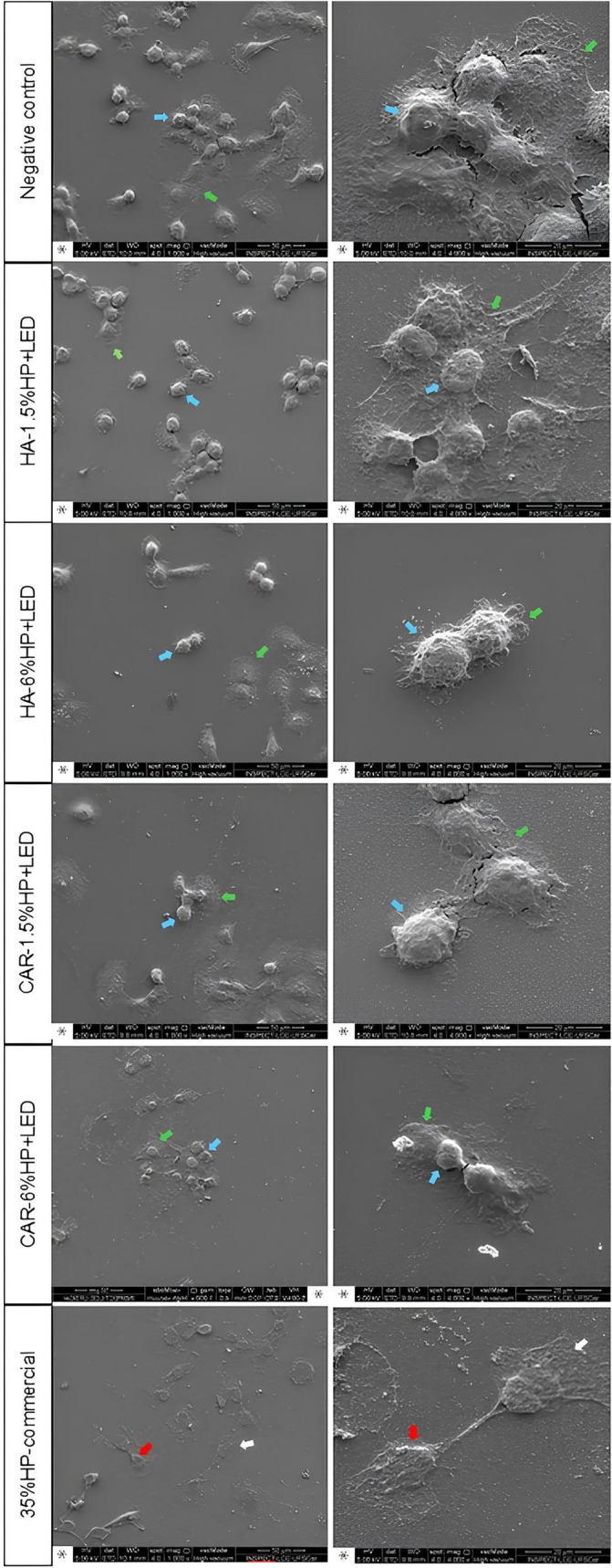
SEM images of MDPC‐23 cells subjected to trans‐amelodentinal diffusion at magnifications of 1000× and 4000×. Blue arrows indicate cells with extensive cytoplasm and spherical morphology; green arrows point to membrane and cytoplasmic extensions of the cells on the glass substrate; red arrows signify cells with significant morphological changes, and white arrows indicate cell membrane rupture.

The SEM images (Figure 6) reveal that the groups exposed to 1.5% and 6% H_2_O_2_ with particle addition and LED irradiation displayed cells with an extended cytoplasm, spherical morphology (blue arrow), and cytoplasmic membrane extensions (green arrow) on the glass substrate. This was consistent across both CAR and HA groups, regardless of the H_2_O_2_ concentration, resembling the untreated negative control group. In contrast, in the groups bleached with 35% HP‐commercial, extensive areas of the glass substrate were exposed, with only a few cells remaining adhered after exposure to the extracts. Furthermore, significant morphological changes (red arrows) and cell membrane rupture (white arrows) were observed in these cells.

## 4 Discussion

Based on the results, the first research hypothesis was rejected, and the second research hypothesis was accepted. Regarding the first research hypothesis, the bleaching efficacy of the experimental gels did not show a statistically significant difference compared with the commercial bleaching gel containing 35% H_2_O_2_. However, an exception was observed for the experimental gel CAR‐1.5%HP + LED, which demonstrated significantly lower bleaching efficacy than the other experimental gels and the commercial product, specifically in the whiteness index (ΔWI_D_) analysis.

The results of ΔE_00_ indicate that the experimental gels were effective in altering tooth color, regardless of the thickener used or the concentration of H_2_O_2_. Although there was no statistical difference among the groups, the bleaching gel that exhibited the highest ΔE_00_ value was CAR‐6%HP + LED (ΔE_00_ = 6.69), which was comparable to the 35%HP‐commercial (ΔE_00_ = 6.10). Kury et al. [32] investigated the combination of NF_TiO_2_ nanoparticles with violet LED in bleaching gels, and their findings align with the results of the present study. Moreover, their data indicate that gels irradiated with violet LED exhibited significantly higher ΔE_00_ values compared with those that were not irradiated, thereby enabling a reduction of up to five times in the hydrogen peroxide concentration. The ΔE_00_ values in this study exceed both the acceptability (1.8) and perceptibility (0.8) thresholds [1], indicating a clinically significant color change in dental bleaching.

Similar to the ΔE_00_ results, the ΔWI_D_ values were above the acceptability (2.62) and perceptibility (0.72) thresholds [2]. However, in this case, the CAR‐1.5% HP + LED group presented the lowest ΔWI_D_ value and was the only group that statistically differed from the other experimental groups and the 35%HP‐commercial control group. This finding highlights the influence of the thickener on the bleaching efficacy, as previously reported in the study by Guerra Silva et al. [24], where different thickeners for at‐home bleaching were utilized, and significant differences in results were observed.

The ΔE_00_ and ΔWI_D_ values obtained in this study indicate that the incorporation of NF_TiO_2_ nanoparticles into an HA thickener allowed for a large reduction of hydrogen peroxide concentration (1.5% and 6%). However, this was only possible under irradiation with violet LED, which has a wavelength capable of interacting with the nanoparticles [32]. In this manner, the electrons in the valence band (ground state) are excited to the conduction band (excited state). Subsequently, the vacancy left by the electrons in the valence band, now positively charged, recombines with free electrons in the conduction band, resulting in the release of heat or light. If the electrons do not recombine, they can generate positive and negative charges that participate in redox reactions on the nanoparticle surface, leading to the formation of longer‐lived ROS [34, 35].

Although titanium dioxide (TiO_2_) is widely used in healthcare, its cytotoxicity has been highly debated in the scientific community, especially when transformed into nanoparticles (NPs), due to its peculiar characteristics such as size, surface area, and reactivity [28, 29]. Ghosh et al. [27] report that titanium dioxide nanoparticles (TiO_2_) do not compromise cell membrane integrity. However, it has been observed that they decrease the activity of dehydrogenases in human lymphocyte mitochondria, which may lead to the induction of apoptosis and DNA damage. The cytotoxic effects of NPs are time‐and dose‐dependent [30]. In this study, a very low dose of NF_TiO_2_ (5 wt%) was used, and the protocol consisted of three sessions, spaced 7 days apart, with each session lasting only 30 min. This study did not focus on the NPs of NF_TiO_2_ as a specific factor for investigation, making it challenging to assess the cytotoxicity attributed solely to the NPs. However, based on the aforementioned facts, it is assumed that the cytotoxicity caused by them was minimal.

According to the literature, the trans‐amelodentinal diffusion capacity of H_2_O_2_ during the bleaching procedure is related to the physicochemical characteristics of the gel, such as pH^3^, viscosity [25], H_2_O_2_ concentration, application frequency, and exposure time to the dental surface [10]. This study standardized experimental gels to a pH of 6.0 to prevent enamel demineralization and minimize the impact on H_2_O_2_ diffusion into the pulp. The low concentrations of H_2_O_2_ used may have been the cause of the statistical differences observed, as the gels HA‐1.5% HP + LED and CAR‐1.5% HP + LED exhibited the least diffusion. De Oliveira Ribeiro et al. [21] evaluated low‐concentration bleaching gels (10% H_2_O_2_) containing manganese oxide (MnO) particles, a transition metal, as a catalyst for low H_2_O_2_ concentrations, and observed excellent results. They suggested that the mineral phase of oxide could have catalyzed the H_2_O_2_ through a Fenton‐like reaction, explaining the high generation of hydroxyl radicals (OH^−^). It is believed that this same explanation is plausible for the results obtained with the experimental gels in this study, which contained NF_TiO_2_ NPs, also a transition metal, as a catalyst.

Excess ROS can induce signaling pathways that lead to apoptosis (programmed cell death) or necrosis (disordered cell death), resulting in a reduced number of viable cells [12]. Moreover, studies have shown that the viability of MDPC‐23 cells is inversely proportional to the level of oxidative stress [17, 18], which corroborates the findings of this study.

The oxidative stress analysis revealed that groups containing the lowest H_2_O_2_ concentrations (HA‐1.5% HP + LED and CAR‐1.5% HP + LED) exhibited the lowest oxidative stress levels, with cell viability approaching that of the negative control group, which was not bleached. Although the groups with 6% H_2_O_2_ showed satisfactory results, they were statistically different from the groups with the lower concentration of H_2_O_2_ in the cell viability analysis. The 35% HP‐commercial group, on the other hand, statistically differed from all experimental groups, showing extremely high oxidative stress values and low cell viability. These results confirm the findings of Kury et al. [20], who observed improved aesthetic efficacy and reduced cytotoxicity when NF_TiO_2_ NPs were added to the bleaching gel, associating low concentrations of H_2_O_2_ (6%) with violet LED irradiation.

Oxygen is a highly reactive molecule and can be partially reduced, resulting in the formation of various reactive chemical agents. Through the process of electron transfer or energy absorption, ROS are produced, including superoxide radical (O_2_^−^) and hydroxyl radical (OH^−^), among others. While NF_TiO_2_ nanoparticles do not directly damage pulp cells, their role in ROS generation within the bleaching gel makes them a critical component in modulating oxidative stress, which, according to the literature, is primarily responsible for causing damage to cells [12, 13]. Based on the results of this study, the second research hypothesis was accepted. It is hypothesized that the interaction of NF_TiO_2_ nanoparticles with violet LED accelerated the production of ROS with a longer half‐life, allowing low concentrations of hydrogen peroxide to achieve the same bleaching efficacy as high‐concentration gels while decreasing cytotoxicity based on the concentration used.

The SEM analysis revealed that the groups exposed to 1.5% and 6% hydrogen peroxide, combined with NF_TiO_2_ nanoparticles and irradiated with LED, exhibited cells with extensive cytoplasm, spherical morphology, and extensions of the membrane and cytoplasm, characteristics indicative of preserved cellular integrity. This behavior can be explained by the low concentrations of hydrogen peroxide present in the gels, which had their action enhanced by the photocatalysis of NF_TiO_2_ nanoparticles. As previously reported, when irradiated with violet LED, the nanoparticles promote the excitation of electrons from the valence band to the conduction band, generating electron–hole pairs that, instead of causing direct damage to the cell membrane, facilitate the formation of ROS [34, 35]. ROS, at moderate concentrations, can induce oxidative effects that are sufficiently low to prevent membrane rupture, thereby preserving its integrity. In contrast, in the 35%HP‐commercial group, the high concentration of hydrogen peroxide and the absence of this modulation by nanoparticles resulted in greater generation of ROS, leading to severe oxidative damage to the cell membrane, observed as extensive areas of exposed substrate and cell rupture. This suggests that the combination of low concentrations of hydrogen peroxide with NF_TiO_2_ nanoparticles not only maintains bleaching efficacy but also plays a crucial role in mitigating oxidative damage to cells, protecting the cell membrane from ruptures induced by elevated oxidative stress.

Among the experimental groups, the bleaching gel with 1.5% H_2_O_2_ and hyaluronic acid (HA‐1.5% HP + LED) demonstrated bleaching efficacy comparable to the 35% HP‐commercial, as indicated by the ΔWI_D_ analysis, with no statistically significant difference between them. In contrast, the CAR‐1.5% HP + LED group did not achieve the same bleaching efficacy. This suggests that hyaluronic acid can optimize bleaching performance, presenting a viable alternative to carbomer‐based formulations. SEM analysis further revealed a higher density of adherent cells with preserved morphology, including well‐defined cytoplasmic extensions in the HA‐1.5% HP + LED group, indicative of enhanced cell viability and proliferation. These findings highlight hyaluronic acid's potential as an effective bioactive thickener in bleaching gels, maintaining aesthetic efficacy and minimizing cytotoxicity compared with traditional thickeners.

Although the results of this in vitro cytotoxicity study are promising, it is important to recognize that in vitro models cannot fully replicate the complexities and interactions that occur in a live biological environment, such as immune responses and tissue regeneration. To overcome these limitations, future studies should include in vivo tests to validate the safety and efficacy of bleaching gels containing NF_TiO_2_ nanoparticles in clinically relevant settings. Such studies could provide a more comprehensive understanding of the long‐term effects and confirm the biocompatibility of the developed materials. Thus, the incorporation of NF_TiO_2_ nanoparticles in bleaching gels with low concentrations of hydrogen peroxide may not only maintain bleaching efficacy but also significantly reduce cytotoxicity, paving the way for the formulation of safer and more effective bleaching agents for clinical use.

## 5 Conclusion

Within the limitations of this study, it was concluded that the experimental bleaching gels incorporating hyaluronic acid, co‐doped titanium dioxide nanoparticles, and low concentrations of hydrogen peroxide, when irradiated with violet LED, achieved the same bleaching efficacy as commercial gels containing 35% hydrogen peroxide while demonstrating no significant cytotoxicity to MDPC‐23 pulp cells.

## Disclosure

The authors have nothing to report.

## Conflicts of Interest

The authors declare no conflicts of interest.

## Data Availability

Research data are not shared.
